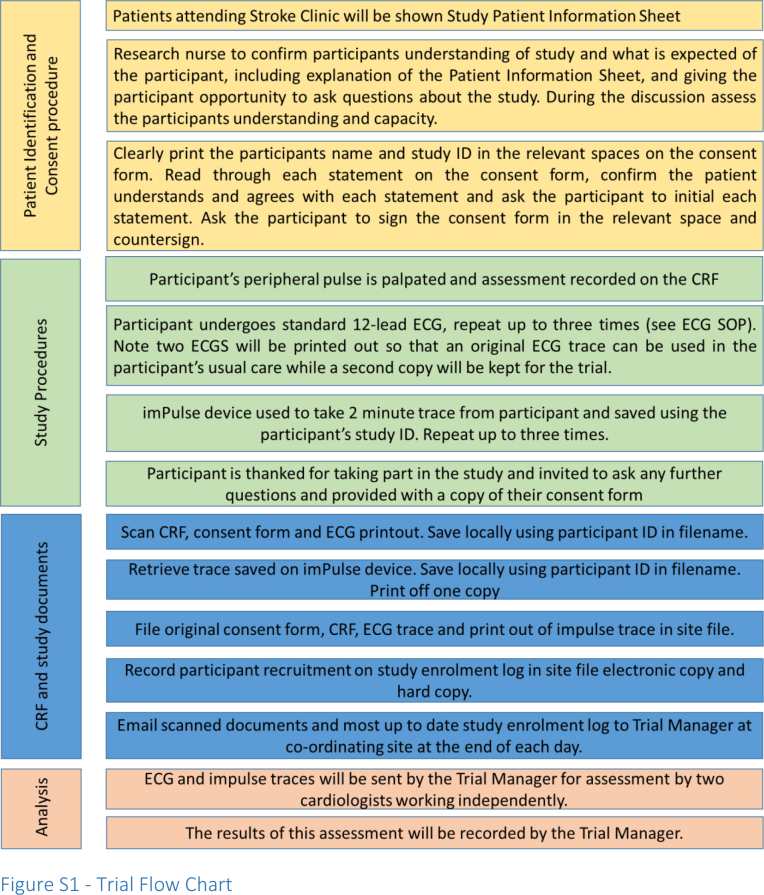# Performance of the imPulse device for the detection of atrial fibrillation in hospital settings

**DOI:** 10.1016/j.cvdhj.2022.05.002

**Published:** 2022-05-26

**Authors:** Sinéad T.J. McDonagh, Shelley Rhodes, Fiona C. Warren, Sam Keenan, Claire Pentecost, Philip Keeling, Martin James, Rod S. Taylor, Christopher E. Clark

**Affiliations:** ∗ Primary Care Research Group, Exeter, United Kingdom; † Exeter Clinical Trials Unit, College of Medicine and Health, University of Exeter Medical School, Exeter, United Kingdom; ‡ Royal Devon and Exeter National Health Service Foundation Trust, Exeter, United Kingdom; § Torbay and South Devon NHS Foundation Trust, Torquay, United Kingdom; ‖ MRC/CSO Social and Public Health Sciences Unit & Robertson Centre for Biostatistics, Institute of Health and Wellbeing, University of Glasgow, Glasgow, Scotland

**Keywords:** Atrial fibrillation, Screening, Arrhythmia, Sensitivity, Specificity, Diagnostics

## Abstract

**Background:**

Atrial fibrillation (AF) increases thromboembolism and stroke risk; this can be reduced by oral anticoagulation, but only if AF is detected. A portable, point-of-care device, capable of accurately detecting and identifying AF, could reduce workload and diagnostic delay by minimizing need for follow-up 12-lead electrocardiogram (ECGs).

**Objective:**

To assess the diagnostic performance of the Plessey imPulse lead I ECG device compared with a 12-lead ECG in detecting AF.

**Methods:**

Cross-sectional diagnostic accuracy study. Participants underwent simultaneous 12-lead ECG and imPulse device recordings. The imPulse device reports AF to be “probable,” “possible,” “unlikely,” or “uncontrolled AF unlikely.” imPulse and ECG reference results were cross-tabulated; sensitivity, specificity, positive/negative predictive values, and positive/negative likelihood ratios with 95% confidence interval (CI) were estimated based on different imPulse device report categorizations and heart rate subgroups.

**Results:**

A total of 217 participants were recruited (mean age 70.2 [standard deviation 12.7]), 56% male, 57% outpatients, 43% inpatients) and 199 were included in analyses. AF was diagnosed on ECG for 41 of 199 (20.6%) participants and reported by imPulse as possible, probable, or uncontrolled AF unlikely present for 49 of 199 (24.6%). Sensitivity and specificity for imPulse detection of possible, probable, or uncontrolled AF unlikely vs unlikely, compared with ECG, were 80.5% (95% CI, 65.1%–91.2%) and 89.9% (84.1%–94.1%), respectively. When probable or uncontrolled AF unlikely were compared vs possible or unlikely AF, sensitivity and specificity were 63.4% (46.9%–77.9%) and 98.1% (94.6%–99.6%), respectively.

**Conclusion:**

The imPulse device has moderate sensitivity and good specificity compared with ECG AF detection in a hospital setting.

**Key Findings**

• In this study, set in 1 hospital, the imPulse lead I electrocardiogram (ECG) device (Plessey Semiconductors Ltd, Plymouth, Devon, UK) was compared with simultaneous ECG traces for the detection of atrial fibrillation (AF).
• The sensitivity and specificity for AF detection by the imPulse device, compared with ECG, were 80.5% (95% confidence interval, 65.1%–91.2%) and 89.9% (84.1%–94.1%), respectively, when comparing the possible, probable, and uncontrolled AF unlikely categories against unlikely AF.
• Where probable or uncontrolled AF unlikely were compared with possible or unlikely AF, sensitivity and specificity were 63.4% (46.9.9%–77.9%) and 98.1% (94.6%–99.6%).
• The imPulse device performs best at higher heart rates, as reflected in the improved sensitivity and specificity values in heart rates over 80 beats per minute.
• The imPulse device was easy to use and acceptable to both patients and clinicians.

## Introduction

Atrial fibrillation (AF) is the most common cardiac arrhythmia worldwide.^1^ Global prevalence is increasing and it represents a major health burden.^2^ AF is prevalent in around 2% of the general population^3^; it carries a 5-fold increased risk of thromboembolism and stroke, compared to those in sinus rhythm.^4^ Paroxysmal AF carries a similar risk of ischemic stroke to that from sustained AF^5^; this has heightened interest in detecting all forms of undiagnosed AF.

Approximately 15% of all strokes are directly attributable to AF; this risk can be significantly reduced through earlier AF detection and treatment with oral anticoagulants.^6^ AF is also a major contributor to cognitive decline in the elderly.^7^ Therefore, screening for AF, particularly in older populations, is an important intervention to reduce future stroke and cognitive impairment.^7^

Many people with AF are asymptomatic, so clinical detection of arrhythmias depends on opportunistic palpation of an irregular pulse.^8^, ^9^, ^10^ A regular pulse has a high negative predictive value for AF (99%–100% of people with a regular pulse do not have AF); however, the positive predictive value of an irregular pulse is only approximately 12%.^11^ Therefore, detection of an irregular pulse usually necessitates booking a further appointment in primary care to record and interpret an electrocardiogram (ECG) and confirm AF presence.

Handheld ECG-like devices could improve opportunistic screening, particularly if clinicians trained in accurate interpretation of ECGs are unavailable, by offering point-of-care diagnosis without additional appointments for ECGs.^12^ Single-lead ECG devices, the MyDiagnostick (Applied Biomedical Systems BV, Maastricht, The Netherlands) and the AliveCor (Mountain View, CA), have shown promise as AF screening tools and could also be used to better target the requirement for more time-consuming 12-lead ECGs. Both devices successfully detected AF, although manual interpretation of their traces proved more specific, but less sensitive, than their algorithms.^13^^,^^14^

Development of portable, wireless ECG devices that can be used at point of care by members of primary or community care teams could have a significant impact on health outcomes. Such devices could improve direct detection rates of AF, thus potentially reducing stroke incidence in at-risk populations, by identifying people who can benefit immediately from anticoagulation. The use of these devices should also reduce workload for primary care staff by minimizing the number of confirmatory negative ECGs required.

**Figure 1:**
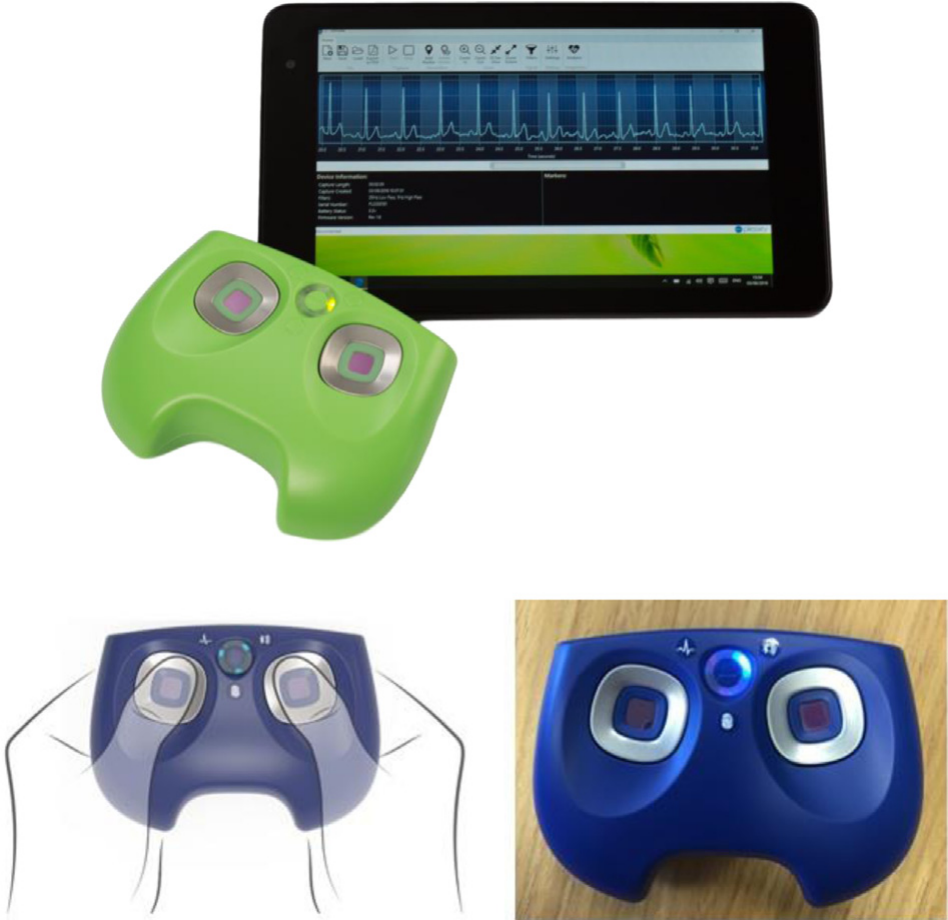
Appearance and mode of use of the imPulse device and tablet (Plessey Semiconductors Ltd, Plymouth, Devon, UK).

The Plessey imPulse device (Plessey Semiconductors Ltd, Plymouth, Devon, UK) is a new mobile lead I ECG device designed to detect AF (Figure 1). This study was undertaken to determine the sensitivity and specificity of this device in comparison to pulse palpation and a reference standard 12-lead ECG for AF diagnosis in a hospital setting.

## Methods

We undertook a prospective diagnostic study to evaluate the performance of the Plessey imPulse device as an AF screening tool. The study was designed according to the Standards for Reporting of Diagnostic Accuracy (STARD)^15^ and approved by the National Health Service (NHS) Research Ethics Committee and Health Research Authority: IRAS Number 189891; MREC Ref 18/NW/0421. The study was registered at ClinicalTrials.gov, identifier: NCT03524625.

### Participants

Participants were initially recruited consecutively from the stroke clinic at the Royal Devon & Exeter Hospital NHS Foundation Trust, a large district general hospital in Devon, England. To reach the planned sample size, recruitment was later extended to the acute stroke and cardiology wards and 1 cardioversion clinic. Participants were NHS patients, aged over 18 years, who were able to hold the imPulse device with both hands and provide informed consent to participate in the study. The presence of an artificial pacemaker or cardioverter-defibrillator was the only exclusion criterion. Data were collected from July 2018 until August 2019.

### The imPulse device

imPulse is a handheld device equipped with an AF-specific detection algorithm, 2 touch sensors, and a programmable electrical medical system that directly transmits real-time trace recordings via Bluetooth to a viewer software application on any Windows-compatible device (Figure 1). The imPulse algorithm firstly analyzes the trace and classifies it as probable or possible AF or neither (ie, not AF). Secondly, these classifications are reported in 1 of 4 ways according to underlying heart rate: (1) probable AF detected; (2) possible AF detected; (3) uncontrolled AF is unlikely (ie, AF is detected but considered controlled owing to recorded heart rate <70 beats per minute); or (4) AF is unlikely (Table 1).

**Table 1:** Summary of imPulse output statements

	Trace interpretation	Heart rate (beats/min)	imPulse output statement
1	AF probable	≥80	Probable atrial fibrillation detected
2	AF possible or AF probable	≥70	Possible atrial fibrillation detected
3	AF probable	<70	Uncontrolled atrial fibrillation is unlikely†
4	AF neither probable nor possible	Any	Atrial fibrillation is unlikely

AF = atrial fibrillation.

† Indicating that AF is detected but considered controlled owing to recorded heart rate.

### Test methods

Research nurses trained in operation of the imPulse device, peripheral pulse palpation, and 12-lead ECG recording collected demographic data from patients after obtaining informed consent. Following a standard protocol, participants were prepared for the ECG resting on a couch, and the peripheral pulse was palpated. Participants then held the imPulse device in both hands, with each thumb pressed against the sensors (Figure 1). The imPulse recording was taken over a 2-minute period, during which a contemporaneous 15-second 12-lead ECG trace was also recorded. Up to 3 attempts to record a satisfactory ECG were made. Study conduct is summarized in Supplemental Figure S1.

Reference standard 12-lead ECGs were analyzed independently, after completion of data collection, by 2 clinicians experienced in ECG analysis (CEC and AR). Clinicians were blinded to the imPulse output; each provided an ECG report, stating whether the patient was in sinus rhythm or AF, and noted any other abnormalities present. Disagreements in interpretation were resolved by a third clinician (PK), where necessary.

### Sample size

Hospital-based AF prevalence was estimated to be 20% (unpublished clinic data). Assuming sensitivity and specificity of the device to be 80%, precision errors based on 95% confidence intervals (CI) were estimated to be ±16% and ±7%, respectively, for a sample size of 200. After the first 100 participants were recruited, AF prevalence (in stroke clinics) was checked and was lower than anticipated (12%); hence, additional participants were sought from acute stroke and cardiology wards and a cardioversion clinic to achieve the planned overall AF prevalence of 20%.

### Analysis

Results from the imPulse (index test) and ECG (reference standard) were cross-tabulated and sensitivity, specificity, and positive and negative predictive values calculated with corresponding 95% CI. To conduct these analyses, the 4 potential outputs of the imPulse device were collapsed into logical groups to give 2 × 2 comparison tables. The study was powered regardless of underlying heart rate; therefore, the 2 primary comparisons were (1) the comparison of AF probable, AF possible, or uncontrolled AF unlikely with AF unlikely (comparison A); and (2) the comparison of AF probable or uncontrolled AF unlikely with AF possible or AF unlikely (comparison B; Table 1). Since the device algorithm output statements take account of underlying heart rate (Table 1), a series of logical subgroup analyses according to heart rate (including any, ≥80, <80, ≥70, <70 beats per minute) were performed as secondary outcomes (Supplemental Table S1).

Likelihood ratios were used to calculate positive and negative predictive values for AF diagnosis in each comparison. Participants with indeterminate or missing results on index or reference measures were excluded from the primary analysis. No imputation of missing data was performed. Participant comments on the concept and utility of the device were sought after each investigation.

### Patient and public involvement

Patient representatives were involved in the production of the Patient Information Sheet, production of the plain English summary of the research, and dissemination to end users, including patients with arrhythmias and clinicians who may use the device for opportunistic AF screening.

## Results

**Figure 2:**
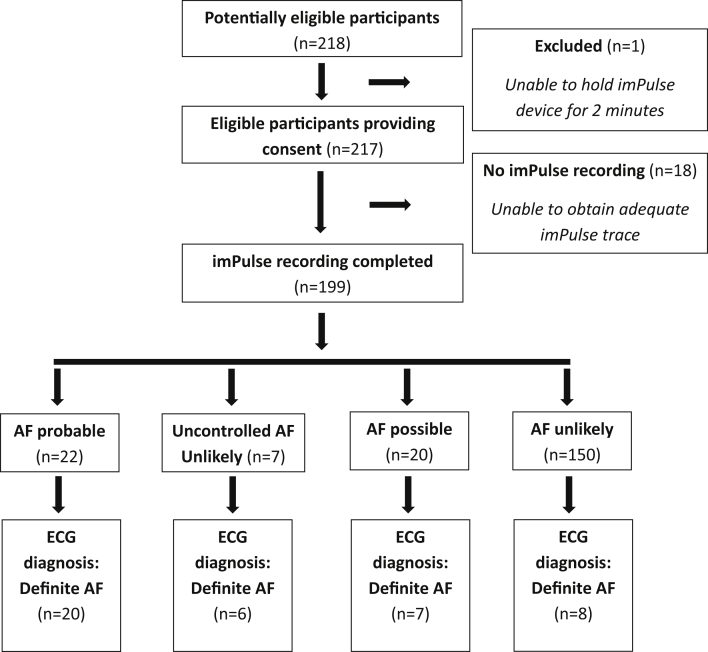
Flow of participants in study. AF = atrial fibrillation; ECG = electrocardiogram.

Between July 23, 2018, and August 1, 2019, 218 hospital patients were assessed for eligibility; 217 were eligible and consented to participate. No participants withdrew from the study (Figure 2).

### Participant demographics

Of 217 recruited participants, 74 had hypertension, 33 had type 2 diabetes mellitus, 13 had asthma, 7 had chronic obstructive pulmonary disease, 16 had high cholesterol, 29 had a history of cerebrovascular events, and 28 had had at least 1 myocardial infarction. An ECG was completed for 215 (99%) participants (with 2 ECG traces unsuitable for analyses owing to an uninterpretable noisy signal); of these, 199 (92%) participants had an acceptable simultaneous imPulse recording. Mean age of the 199 participants producing complete data was 70 years (standard deviation 12.7) and 56% were male; characteristics are summarized in Table 2. Of the 199 participants with an imPulse recording, 150 were unlikely to have AF, and 142 of these 150 were confirmed by ECG as not having AF (Figure 2).

**Table 2:** Baseline characteristics: Included participants (n = 199)

Sex, n (%)	
Male	111 (56)
Female	88 (44)
Age, mean (SD), median [min, max]	70.2 (12.7), 71 [28, 93]
Ethnic group, n (%)	
White British	194 (97)
White other	2 (1)
White & Asian	1 (<1)
Pakistani	1 (<1)
Not known	1 (<1)
Participant source, n (%)	
Inpatient	84 (42)
Outpatient	115 (58)
Participant has relevant medical history? n (%)	
Yes	175 (88)
No	24 (12)
Not known	0 (0)
Was participant on concomitant drugs? n (%)	
Yes	86 (43)
No	111 (56)
Not known	2 (1)
Individual drug use, n (%)	
Acebutolol	1 (1)
Atenolol	6 (7)
Bisoprolol	65 (76)
Carvedilol	3 (3)
Digoxin	10 (12)
Diltiazem	2 (2)
Metoprolol	1 (1)
Sotalol	2 (2)
Verapamil	2 (2)

### Diagnostic performance

AF was diagnosed on ECG traces for 41 of 199 (20.6%) participants and reported by imPulse as possible, probable, or uncontrolled AF unlikely for 49 of 199 (24.6%) participants. For the primary outcomes, sensitivity and specificity for AF detection by the device, compared with ECG, were 80.5% (95% CI 65.1%–91.2%) and 89.9% (84.1%–94.1%), respectively, for comparison A (comparing possible, probable, and uncontrolled AF unlikely categories to unlikely AF; Table 3). For comparison B (probable AF or uncontrolled AF unlikely compared to possible or unlikely AF; Table 3), sensitivity was 63.4% (46.9%–77.9%) and specificity 98.1% (94.6%–99.6%).

**Table 3:** Primary outcomes—Comparing imPulse with 12-lead electrocardiogram: Full cohort

	imPulse output		
**Comparison A**			
12-lead ECG	Positive (probable AF/UAFU/possible AF)	Negative (unlikely AF)	Total
Positive (AF)	33	8	41
Negative (non-AF)	16	142	158
Total	49	150	199
Prevalence, %) (95% CI)			20.6 (15.2; 26.9)
Sensitivity, % (95% CI)			80.5 (65.1; 91.2)
Specificity, % (95% CI)			89.9 (84.1; 94.1)
Positive predictive value, % (95% CI)			67.3 (52.5; 80.1)
Negative predictive value, % (95% CI)			94.7 (89.9; 97.7)
Likelihood ratio, positive (95% CI)			8.0 (4.9; 13.0)
Likelihood ratio, negative (95% CI)			0.22 (0.12; 0.41)
**Comparison B**			
12-lead ECG	Positive (probable AF/UAFU)	Negative (possible AF/unlikely AF)	Total
Positive (AF)	26	15	41
Negative (non-AF)	3	155	158
Total	29	170	199
Sensitivity, % (95% CI)			63.4 (46.9; 77.9)
Specificity, % (95% CI)			98.1 (94.6; 99.6)
Positive predictive value, % (95% CI)			89.7 (72.6; 97.8)
Negative predictive value, % (95% CI)			91.2 (85.9; 95.0)
Likelihood ratio, positive (95% CI)			33.4 (10.6; 104.9)
Likelihood ratio, negative (95% CI)			0.37 (0.25; 0.56)

AF = atrial fibrillation; ECG = electrocardiogram; UAFU = Uncontrolled atrial fibrillation unlikely.

### Heart-rate subgroup analysis

Subgroup analyses are summarized in Table 4. For participants with a heart rate ≥80 beats per minute (n = 53), sensitivity for AF detection was 95.2% (76.2%–99.9%) and specificity was 78.1% (60.0%–90.7%) where both probable and possible AF outputs were considered diagnostic of AF (comparison C). Specificity improved to 93.8% (79.2%–99.2%; Supplemental Table S2) when only probable AF was considered diagnostic of AF (comparison D).

**Table 4:** Summary of main findings for all comparisons

Comparison	Description	HR	N	AF prevalence	Sensitivity	Specificity	PPV	NPV
A	Probable AF/UAFU/possible AF vs unlikely	Any	199	20.6 (15.2; 26.9)	80.5 (65.1; 91.2)	89.9 (84.1; 94.1)	67.3 (52.5; 80.1)	94.7 (89.9; 97.7)
B	Probable AF/UAFU vs possible AF/unlikely AF	Any	199	20.6 (15.2; 26.9)	63.4 (46.9; 77.9)	98.1 (94.6; 99.6)	89.7 (72.6; 97.8)	91.2 (85.9; 95.0)
C	Probable AF/possible AF vs unlikely AF	≥80	53	39.6 (26.5; 54.0)	95.2 (76.2; 99.9)	78.1 (60.0; 90.7)	74.1 (53.7; 88.9)	96.2 (80.4; 99.9)
D	Probable AF vs possible AF/unlikely AF	≥80	53	39.6 (26.5; 54.0)	95.2 (76.2; 99.9)	93.8 (79.2; 99.2)	90.9 (70.8; 98.9)	96.8 (83.3; 99.9)
E	Possible AF/UAFU vs unlikely AF	<80	146	13.7 (8.6; 20.4)	65.0 (40.8; 84.6)	92.9 (86.9; 96.7)	59.1 (36.4; 79.3)	94.4 (88.7; 97.7)
F	Probable AF/possible AF vs unlikely AF	≥70	106	27.4 (19.1; 36.9)	93.1 (77.2; 99.2)	80.5 (69.9; 88.7)	64.3 (48.0; 78.4)	96.9 (89.2; 99.6)
G	Probable AF vs possible AF/unlikely AF	≥70	106	27.4 (19.1; 36.9)	69.0 (49.2; 84.7)	97.4 (90.9; 99.7)	90.9 (70.8; 98.9)	89.3 (80.6; 95.0)
H	UAFU vs unlikely AF	<70	93	12.9 (6.8; 21.5)	50.0 (21.1; 78.9)	98.8 (93.3; 100)	85.7 (42.1; 99.6)	93.0 (85.4; 97.4)
	Peripheral pulse palpation vs ECG	Any	215	20.9 (15.7; 27.0)	93.3 (81.7; 98.6)	86.5 (80.4; 91.2)	64.6 (51.8; 76.1)	98.0 (94.3; 99.6)
	Peripheral pulse palpation vs ECG	≥80	53	39.6 (26.5; 54.0)	100.0 (83.9; 100.0)	81.3 (63.6; 92.8)	77.8 (57.7; 76.1)	100.0 (86.8; 100.0)
	Peripheral pulse palpation vs ECG	<80	146	13.7 (8.6; 20.4)	85.0 (62.1; 96.8)	88.1 (81.1; 93.2)	53.1 (34.7; 70.9)	97.4 (92.5; 99.5)

Note. This table summarizes the AF prevalence, sensitivity, specificity, positive predictive values, and negative predictive values based on outputs from the imPulse device (Plessey Semiconductors Ltd, Plymouth, Devon, UK), peripheral pulse palpation, and electrocardiography across a range of different heart rates. More detailed information regarding the comparisons is available in Supplemental Tables 2–6.

AF = atrial fibrillation; ECG = electrocardiogram; HR = heart rate; NPV = negative predictive value; PPV = positive predictive value; UAFU = uncontrolled AF unlikely.

With a heart rate <80 beats per minute (n = 146), sensitivity and specificity for AF detection were 65.0% (40.8%–84.6%) and 92.9% (86.9%–96.7%), respectively (Supplemental Table S3).

For participants with a heart rate >70 beats per minute (n = 106), sensitivity for detection of AF was 93.1% (77.2%–99.2%) and specificity was 80.5% (69.9%–88.7%) where both probable and possible AF outputs were considered diagnostic of AF (comparison F; Supplemental Table S4). Where only probable AF was considered diagnostic of AF (comparison G, n = 106), sensitivity fell to 69.0% (49.2%–84.7%) and there was a modest rise in specificity to 97.4% (90.9%–99.7%).

With heart rates <70 beats per minute (n = 93), sensitivity and specificity for detection of AF were 50.0% (21.1%–78.9%) and 98.8% (93.3%–100%), respectively (comparison H; Supplemental Table S5).

Sensitivity of peripheral pulse palpation for the whole cohort in diagnosing AF was 93.3% (81.7%–98.6%) and specificity 86.6% (80.4%–91.2%; Supplemental Table S6). The negative predictive value was higher than the values obtained with the imPulse device; however, the positive predictive value was modest at 64.6% (51.8%–76.1%) and lower than the values obtained with the imPulse device.

### Assessment of acceptability

Participants and clinicians found the use of the imPulse device for detecting AF acceptable; it was considered easy to use and most participants liked the handheld device concept, although some felt that it was “too reminiscent of a toy or games console.”

## Discussion

In a hospital cohort, this diagnostic accuracy study found that the overall sensitivity and specificity of the Plessey imPulse lead I ECG device were 80.5% (65.1%–91.2%) and 89.9% (84.1%–94.1%) respectively, using the possible, probably, and uncontrolled AF unlikely categories for diagnosis in comparison with a 12-lead ECG (comparison A). There was evidence of higher sensitivity in subgroups of participants with heart rates >70 and >80 beats per minute diagnostic thresholds used by the imPulse algorithm. Negative predictive values were high and positive predictive values were superior to pulse palpation. The device was reported to be easy to use.

Current guidelines recommend AF detection by opportunistic screening.^9^ New devices, such as that described here, may improve the feasibility of future AF screening programs by offering improved sensitivity, specificity, and ease of detection in comparison to traditional techniques.^16^ The imPulse device demonstrated moderate sensitivity and good specificity compared with the gold-standard detection of AF using a 12-lead ECG. More specifically, comparison A (possible, probable, and uncontrolled AF unlikely vs unlikely AF) showed superior sensitivity and negative predictive values, but inferior specificity and positive predictive values, compared with comparison B (probable AF or uncontrolled AF unlikely compared to possible or unlikely AF). However, both comparison A and B demonstrated superior specificity and positive predictive values compared with pulse palpation, therefore potentially reducing the need for follow-up confirmatory ECG appointments in non-AF cases, if imPulse is used as a practical alternative to pulse palpation.

Device performance is comparable to, and in some respects superior to, other algorithm-driven devices.^17^^,^^18^ Although this study was undertaken in stroke and cardiology wards, it may also be sensible to suggest that the imPulse device could be considered for AF screening in primary and other secondary care settings, to reduce the number of ECG referrals, burden on healthcare professionals, cost to the NHS, and time taken to diagnose AF. Atrial flutter is sometimes excluded from diagnostic accuracy studies; this seems illogical, since both AF and atrial flutter are associated with similar stroke risks, and with each other.^9^^,^^19^ The current study included people with atrial flutter in all analyses. Both the device and peripheral pulse palpation perform less well at lower heart rates. However, the imPulse device demonstrated superior specificity to pulse palpation, with higher positive predictive values, thus suggesting the potential to reduce the need for confirmatory ECGs in non-AF cases, if used instead of pulse palpation.

Alternative AF detection technologies exist. There are a range of devices without algorithms, which require clinician interpretation of lead I recordings.^17^ These are not suited to community screening programs owing to the requirements and associated costs of a qualified clinician to interpret outputs in real time. Similarly, some oscillometric sphygmomanometers incorporate AF detection algorithms.^20^ A recent systematic review assessed the diagnostic accuracy of 6 blood pressure–based devices for opportunistic detection of AF.^11^ All 7 studies on these 6 devices gave specificity values >85% and sensitivity values >90%, favorably comparing with manual pulse palpation. With each study having differing populations, no assessment of either positive or negative predictive values was given for these devices, in a screening population. Such devices have been reported to be cost saving owing to superior sensitivity and specificity compared to pulse palpation, and trialed in detection of AF in community settings^21^^,^^22^; however, any detection of pulse irregularity requires ECG confirmation before anticoagulation can be considered.^9^ New wearable technology, such as the Apple Watch with contemporaneous app-based AF detection software (KardiaBand), offers further possibilities for diagnosis in individuals, but such devices are not designed for mass screening. Current data suggest a lower specificity for the Apple Watch device in comparison with the other devices considered here, and the Apple Watch/KardiaBand system was unable to interpret one-third of recordings.^18^

Previous reports of other algorithm-based devices (for example, the MyDiagnostick and AliveCor) have not reported population heart rates or rate-dependent sensitivity analyses, so it is not known whether they may also perform differently across different heart rates.^13^^,^^19^^,^^23^^,^^24^ Based on our findings, future diagnostic accuracy studies for AF detection should take underlying heart rate into account, and be adequately powered to examine performance in subgroups according to heart rate; it is important that the contribution of heart rate to diagnostic algorithms, as we have described here for the ImPulse device, is clearly stated.

### Limitations

This is the first formally powered study of AF diagnostic accuracy for the Plessey imPulse device. We planned to recruit 200 individuals to participate in the study; however, 18 participants that completed the study were not included in the primary analyses owing to poor signal on the imPulse device and subsequent unusable recordings, despite careful adherence to the study protocol. Further recruitment via cardiology wards and cardioversion clinics was undertaken to achieve full data on 199 participants. By powering our analyses on a background AF prevalence of 20%, the current findings may be more relevant to other non–specialist hospital settings than previous studies of other devices. For example, the MyDiagnostick device was studied in more selected population samples, with 53% AF prevalence, and the AliveCor device was studied in a geriatric hospital inpatient population, with 36% AF prevalence noted.^13^^,^^24^ Diagnostic accuracy has also been reported for the Apple Watch with KardiaBand in a cohort with AF presenting before and after cardioversion (effectively, 100% AF prevalence).^18^

Only 1 previous diagnostic accuracy study (for a clinician-interpreted device) has been conducted with simultaneous recording of device and reference ECG traces.^25^ Our study has further confirmed that simultaneous data acquisition of device and 12-lead ECGs is feasible, and sets a reference standard for the conduct of future studies, by avoiding any risk of bias introduced by timing issues or delays between ECG and device recordings.

## Conclusion

Compared with simultaneous measurement with a reference gold-standard 12-lead ECG method, the Plessey imPulse device has moderate sensitivity and good specificity, and high negative predictive values and positive predictive values, in a hospital cohort. The imPulse device also reported superior positive predictive values compared with pulse palpation. The findings are consistent with other lead I devices. Sensitivity analyses showed that baseline heart rate may play a role in the AF diagnostic accuracy of the imPulse device, with improved sensitivity reported at higher compared with lower heart rates. Underlying heart rate should be considered in future diagnostic accuracy studies. The findings from this study suggest that further work to evaluate the clinical performance and cost effectiveness of the imPulse device in primary care detection of asymptomatic AF are warranted to determine the role of this and other devices in future opportunistic AF screening programs.

## Figures and Tables

**Supplemental Figure S1:**