# Supplementary material for: Performance of the imPulse device for the detection of atrial fibrillation in hospital settings
Source: Cardiovasc Digit Health J. 2022 May 26;3(4):171–8. doi: 10.1016/j.cvdhj.2022.05.002 (PMC9422064; doi:10.1016/j.cvdhj.2022.05.002)
Supplement: Supplementary Tables S1-S6 [file mmc1.docx]

# Supplementary data

## Table S1 – summary of logical subgroup analyses according to heart rate

| Heart rate (beats per minute) | Comparison | Subgrouping* |
| --- | --- | --- |
| heart rate ≥80 | C | output 1 or 2 compared to output 4 |
|  | D | output 1 compared to output 2 or 4 |
|  |  |  |
| heart rate <80 | E | outputs 2 or 3 compared to output 4 |
|  |  |  |
| heart rate ≥ 70 | F | output 1 or 2 compared to output 4 |
|  | G | output 1 compared to output 2 or 4 |
|  |  |  |
| heart rate <70 | H | output 3 compared to output 4 |
|  |  |  |
| *output 1 = Probable atrial fibrillation detected; 2 = Possible atrial fibrillation detected; 3 = Uncontrolled atrial fibrillation is unlikely (i.e. AF is detected but heart rate <70 beats per minute so judged to be controlled); 4 = Atrial fibrillation is unlikely | | |

## Table S2 - Comparison for participants with heart rate 80 beats per minute or above

|  | imPulse output | | |
| --- | --- | --- | --- |
| **Comparison C** | | | |
| 12-lead ECG | Positive (probable AF/possible AF) | Negative (unlikely AF) | Total |
| Positive (AF) | 20 | 1 | 21 |
| Negative (non-AF) | 7 | 25 | 32 |
| Total | 27 | 26 | 53 |
|  | | | |
| Prevalence (%) (95% CI) | | | 39.6 (26.5; 54.0) |
| Sensitivity (%) (95% CI) | | | 95.2 (76.2; 99.9) |
| Specificity (%) (95% CI) | | | 78.1 (60.0; 90.7) |
| Positive predictive value (%) (95% CI) | | | 74.1 (53.7; 88.9) |
| Negative predictive value (%) (95% CI) | | | 96.2 (80.4; 99.9) |
| Likelihood ratio (positive) (95% CI) | | | 4.4 (2.3; 8.4) |
| Likelihood ratio (negative) (95% CI) | | | 0.06 (0.01; 0.42) |
|  | | | |
| **Comparison D** | | | |
| 12-lead ECG | Positive (probable AF) | Negative (possible AF/unlikely AF) | Total |
| Positive (AF) | 20 | 1 | 21 |
| Negative (non-AF) | 2 | 30 | 32 |
| Total | 22 | 31 | 53 |
| Sensitivity (%) (95% CI) | | | 95.2 (76.2; 99.9) |
| Specificity (%) (95% CI) | | | 93.8 (79.2; 99.2) |
| Positive predictive value (%) (95% CI) | | | 90.9 (70.8; 98.9) |
| Negative predictive value (%) (95% CI) | | | 96.8 (83.3; 99.9) |
| Likelihood ratio (positive) (95% CI) | | | 15.2 (4.0; 58.5) |
| Likelihood ratio (negative) (95% CI) | | | 0.05 (0.01; 0.34) |
| *AF: atrial fibrillation; ECG: Electrocardiograph* | | | |

## Table S3 - Comparison for participants with heart rate below 80 beats per minute

| **Comparison E** | imPulse output | | |
| --- | --- | --- | --- |
| 12-lead ECG | Positive (UAFU/possible AF) | Negative (unlikely AF) | Total |
| Positive (AF) | 13 | 7 | 20 |
| Negative (non-AF) | 9 | 117 | 126 |
| Total | 22 | 124 | 146 |
|  | | | |
| Prevalence (%) (95% CI) | | | 13.7 (8.6; 20.4) |
| Sensitivity (%) (95% CI) | | | 65.0 (40.8; 84.6) |
| Specificity (%) (95% CI) | | | 92.9 (86.9; 96.7) |
| Positive predictive value (%) (95% CI) | | | 59.1 (36.4; 79.3) |
| Negative predictive value (%) (95% CI) | | | 94.4 (88.7; 97.7) |
| Likelihood ratio (positive) (95% CI) | | | 9.1 (4.5; 18.5) |
| Likelihood ratio (negative) (95% CI) | | | 0.38 (0.21; 0.69) |
| *AF: atrial fibrillation; ECG: Electrocardiograph; UAFU: Uncontrolled atrial fibrillation unlikely* | | | |

## Table S4 - Comparison for participants with heart rate 70 beats per minute or above

|  | imPulse output | | |
| --- | --- | --- | --- |
| **Comparison F** | | | |
| 12-lead ECG | Positive (probable AF/possible AF) | Negative (unlikely AF) | Total |
| Positive (AF) | 27 | 2 | 29 |
| Negative (non-AF) | 15 | 62 | 77 |
| Total | 42 | 64 | 106 |
|  | | | |
| Prevalence (%) (95% CI) | | | 27.4 (19.1; 36.9) |
| Sensitivity (%) (95% CI) | | | 93.1 (77.2; 99.2) |
| Specificity (%) (95% CI) | | | 80.5 (69.9; 88.7) |
| Positive predictive value (%) (95% CI) | | | 64.3 (48.0; 78.4) |
| Negative predictive value (%) (95% CI) | | | 96.9 (89.2; 99.6) |
| Likelihood ratio (positive) (95% CI) | | | 4.8 (3.0; 7.6) |
| Likelihood ratio (negative) (95% CI) | | | 0.09 (0.02; 0.33) |
|  | | | |
| **Comparison G** | | | |
| 12-lead ECG | Positive (probable AF) | Negative (possible AF/unlikely AF) | Total |
| Positive (AF) | 20 | 9 | 29 |
| Negative (non-AF) | 2 | 75 | 77 |
| Total | 22 | 84 | 106 |
|  | | | |
| Sensitivity (%) (95% CI) | | | 69.0 (49.2; 84.7) |
| Specificity (%) (95% CI) | | | 97.4 (90.9; 99.7) |
| Positive predictive value (%) (95% CI) | | | 90.9 (70.8; 98.9) |
| Negative predictive value (%) (95% CI) | | | 89.3 (80.6; 95.0) |
| Likelihood ratio (positive) (95% CI) | | | 26.6 (6.6; 106.5) |
| Likelihood ratio (negative) (95% CI) | | | 0.32 (0.18; 0.55) |
| *AF: atrial fibrillation; ECG: Electrocardiograph* | | | |

## Table S5 - Comparison for participants with heart rate below 70 beats per minute

| **Comparison H** | imPulse output | | |
| --- | --- | --- | --- |
| 12-lead ECG | Positive (UAFU) | Negative (unlikely AF) | Total |
| Positive (AF) | 6 | 6 | 86 |
| Negative (non-AF) | 1 | 80 | 7 |
| Total | 7 | 86 | 93 |
|  | | | |
| Prevalence (%) (95% CI) | | | 12.9 (6.8; 21.5) |
| Sensitivity (%) (95% CI) | | | 50.0 (21.1; 78.9) |
| Specificity (%) (95% CI) | | | 98.8 (93.3; 100) |
| Positive predictive value (%) (95% CI) | | | 85.7 (42.1; 99.6) |
| Negative predictive value (%) (95% CI) | | | 93.0 (85.4; 97.4) |
| Likelihood ratio (positive) (95% CI) | | | 40.5 (5.3; 307.86) |
| Likelihood ratio (negative) (95% CI) | | | 0.51 (0.29; 0.89) |
| *AF: atrial fibrillation; ECG: Electrocardiograph; UAFU: Uncontrolled atrial fibrillation unlikely* | | | |

## Table S6 - comparison of peripheral pulse palpation with 12-lead ECG

| 12-lead ECG | Peripheral pulse palpation | | |
| --- | --- | --- | --- |
|  | Positive (irregular rhythm) | Negative (regular rhythm) | Total |
| Positive (AF) | 42 | 3 | 45 |
| Negative (non-AF) | 23 | 147 | 170 |
| Total | 65 | 150 | 215 |
|  | | | |
| Prevalence (%) (95% CI) | | | 20.9 (15.7; 27.0) |
| Sensitivity (%) (95% CI) | | | 93.3 (81.7; 98.6) |
| Specificity (%) (95% CI) | | | 86.5 (80.4; 91.2) |
| Positive predictive value (%) (95% CI) | | | 64.6 (51.8; 76.1) |
| Negative predictive value (%) (95% CI) | | | 98.0 (94.3; 99.6) |
| Likelihood ratio (positive) (95% CI) | | | 6.9 (4.7; 10.2) |
| Likelihood ratio (negative) (95% CI) | | | 0.08 (0.03; 0.23) |
| *AF: atrial fibrillation; ECG: Electrocardiograph* | | | |
